# Evaluation of the acquired immune responses to *Plasmodium vivax *VIR variant antigens in individuals living in malaria-endemic areas of Brazil

**DOI:** 10.1186/1475-2875-5-83

**Published:** 2006-10-06

**Authors:** Tatiane R Oliveira, Carmen Fernandez-Becerra, Maria Carolina S Jimenez, Hernando A Del Portillo, Irene S Soares

**Affiliations:** 1 Departamento de Análises Clínicas e Toxicológicas, Faculdade de Ciências Farmacêuticas, Universidade de São Paulo, Av. Prof. Lineu Prestes, 580, 05508-900, São Paulo, SP, Brazil; 2 Departamento de Parasitologia, Instituto de Ciências Biomédicas, Universidade de São Paulo, Av. Prof. Lineu Prestes, 1374, 05508-900, São Paulo, SP, Brazil

## Abstract

**Background:**

The naturally-acquired immune response to *Plasmodium vivax *variant antigens (VIR) was evaluated in individuals exposed to malaria and living in different endemic areas for malaria in the north of Brazil.

**Methods:**

Seven recombinant proteins representing four *vir *subfamilies (A, B, C, and E) obtained from a single patient from the Amazon Region were expressed in *Escherichia coli *as soluble glutathione S-transferase fusion proteins. The different recombinant proteins were compared by ELISA with regard to the recognition by IgM, IgG, and IgG subclass of antibodies from 200 individuals with patent infection.

**Results:**

The frequency of individuals that presented antibodies anti-VIR (IgM plus IgG) during the infection was 49%. The frequencies of individuals that presented IgM or IgG antibodies anti-VIR were 29.6% or 26.0%, respectively. The prevalence of IgG antibodies against recombinant VIR proteins was significantly lower than the prevalence of antibodies against the recombinant proteins representing two surface antigens of merozoites of P. *vivax*: AMA-1 and MSP1_19 _(57.0% and 90.5%, respectively). The cellular immune response to VIR antigens was evaluated by in vitro proliferative assays in mononuclear cells of the individuals recently exposed to *P. vivax*. No significant proliferative response to these antigens was observed when comparing malaria-exposed to non-exposed individuals.

**Conclusion:**

This study provides evidence that there is a low frequency of individuals responding to each VIR antigens in endemic areas of Brazil. This fact may explain the host susceptibility to new episodes of the disease.

## Background

*Plasmodium vivax *is the second most prevalent malaria species of world with an estimated 80–90 million cases a year [1]. In Americas and Asia, *P. vivax *is the most prevalent malaria species, and in Brazil it represents more than 75% of the clinical cases reported annually [2].

Variant antigens exposed on *P. vivax*-infected reticulocytes are encoded by a single multigene superfamily termed *vir *(*P. vivax *variant genes), with circa 600–1,000 copies per haploid genome [3]. Moreover, in silico analysis of *vir *sequences from endemic regions have demonstrated that sequences can be grouped into different subfamilies (A-E) based on sequence similarities and structural properties [4,5]. Furthermore, in silico, analysis has also revealed that *vir *genes are part of the large *pir *superfamily (*Plasmodium *interspersed repeat), conserved among different species and whose members seem to play a major role in antigenic variation [6]. Antigenic variation is a common phenomenon in all species of *Plasmodium *this far studied, including the species infecting rodents, monkeys and humans (*Plasmodium yoelii*, *Plasmodium berghei*, *Plasmodium chabaudi*, *Plasmodium knowlesi, Plasmodium fragile *and *Plasmodium falciparum*) [7]. These *Plasmodium *species apparently use antigenic variation to evade the immune system and to maintain the parasite survival. In *P. falciparum*, variant antigens are implicated in cytoadherence to the endothelium of venullar capillaries in the deep vascular of inner organs. The major role of *vir *genes and their encoding variant proteins in natural infections is presently unknown, although recently it has been proposed that they have a role in spleen-specific cytoadherence and establishment of chronic infections [8].

Several lines of evidence support the idea that antibody responses directed to *P. falciparum *clonally variant surface antigens (VSA) contribute to the acquired immune protection against malaria caused by this protozoan parasite [9-13]. The VSA described to date include *P. falciparum *erythrocyte membrane protein 1 (PfEMP-1) [14] and the rifins [15,16]. Unlike PfEMP1 proteins, VIR proteins are not clonally expressed by individually infected reticulocytes and very little information is available regarding the naturally acquired immune response against these proteins [4]. In order to determine whether VIR proteins are target of naturally acquired immunity, the antibody response of *P. vivax *infected patients in the Brazilian Amazon was recently analysed using glutathione S-transferase fusion proteins (GST-VIR) expressing exon II and representing the various VIR subfamilies (A-E) from three patients [4].

The present study was designed to evaluate the prevalence of IgM, IgG and IgG subclasses to VIR proteins as estimated by ELISA in 200 individuals exposed to malaria from the Amazon Region, Brazil. Seven soluble GST fusion proteins corresponding to four VIR subfamilies (A, B, C, and E) obtained from parasites of a single patient from the Amazon Region were used in this study. The serum recognition pattern of these individuals was compared with their ability to recognize two recombinant proteins representing two merozoite surface antigens of *P. vivax*: the 19 kDa C-terminal region of the Merozoite Surface Protein-1 (MSP1_19_) and the apical membrane antigen 1 (AMA-1). Finally, the study was complemented by estimating in vitro PBMC proliferative responses upon stimulation with these recombinant proteins.

## Methods

### Subjects

After verbal consent, blood samples from 261 individuals were collected. A total of 220 serum samples were used for serological analysis. Two hundred samples were from patients with patent *P. vivax *malaria and 20 from individuals never exposed to malaria (negative controls). Blood samples from *P. vivax *patients were collected in five different malaria endemic regions in the State of Pará, north Brazil: i) city of Belém (n = 64), ii) city of Itaituba (n = 20), iii) city of Marabá (n = 21), iv) city of Tailândia (n = 20), and v) city of Igarapé-Açu (n = 32). Other 43 blood samples were collected in the State of Rondônia, in the north of Brazil. Patent infection was documented by microscopic analysis of Giemsa-stained blood smears. The mean age of this group was 28.3 ± 13.3 years old and 69.7% of the subjects were male. Clinical and laboratory data have been reported elsewhere for all individuals including individuals never exposed to malaria [4,17-19]. Only individuals, from whom precise information on the number of *P. vivax *malaria episodes was available, were used to establish a correlation between the number of malarias and the frequency of responders to recombinant proteins.

Fifteen to twenty ml of venous blood of 41 individuals were collected aseptically in heparinized tubes and transported at room temperature, within 48 h, for lymphoblastic proliferation assays. The first group of individuals was one of healthy adult volunteers from the city of São Paulo, State of São Paulo, in the southeast of Brazil. Malaria is not present in this part of the country and these individuals had never had malaria or traveled to malaria-endemic areas. The total number of volunteers was 14, their average age was 30.1 ± 6.6 years old and 50.0% of the subjects were male. The second group was composed by 27 adults from the area of Augusto Correa, State of Pará in the north of Brazil, and they had been treated for *P. vivax *malaria in the previous 3 months. The mean age of this group was 33.6 ± 15.0 years old and 44.4% of the subjects were male. The Ethics Committee of the University of São Paulo had approved this study.

### Recombinant proteins

#### VIR

Seven glutathione S-transferase fusion proteins corresponding to four *vir *subfamilies (A, B, C, and E) obtained from parasites of a single patient from the Amazon Region were used in this study. The detailed construction has been described elsewhere [4]. Recombinant *Escherichia coli *BL21 (DE3) was grown at 37°C under constant shaking in multiple flasks containing 500 ml of LB-ampicillin. When the preparation reached an OD_600 _= 1.0, isopropyl-β-D thiogalactopyranoside (IPTG, Invitrogen, Auckland, New Zealand) was added to a final concentration of 2 mM, except to VIR-C16, which was induced with 0.1 mM IPTG. Cultures were incubated at 18°C under constant shaking for 16 h and bacterial pellets were obtained by centrifugation and resuspended in sonication buffer [10 mM Tris-HCl pH 8.0, 150 mM NaCl, 1 mM EDTA, 100 μg/ml lysozyme and 0.05 volumes of the Protease Inhibitor Cocktail (Sigma, Saint Louis, USA)]. Bacteria were lysed on ice with the aid of a sonicator (Branson model 450, Danbury, CT). Five sonication cycles, consisting of 30 seconds pulses at 1 min interval, were applied. Bacterial lysates were centrifuged at 3,000 *g *for 30 min at 4°C. Recombinant proteins were purified from the supernatant of the bacterial lysates using Glutathione Sepharose 4B Fast Flow columns (Amersham Biosciences, Uppsala, Sweden), and their purity determined by SDS-PAGE. As control, GST was produced alone.

#### AMA-1

The recombinant protein His_6_-AMA-1 represents AA 43 to 487 of the *P. vivax *AMA-1. This protein was expressed in *E. coli *and purified as previously described [19].

#### MSP1_19_

The recombinant protein His_6_-MSP1_19 _represents the 19 kDa C-terminal region of the MSP-1 (AA 1616–1704, Belém strain). This protein was expressed in *E. coli *and purified as previously described [20].

### Immunoassays

#### Detection of antigen specific IgG antibodies by ELISA

Human IgG antibodies against VIR, AMA-1 and MSP1_19 _were detected by ELISA as described [18,19], except that the plates were coated with 100 ng/well of each protein. The OD_492 _values to each recombinant VIR protein were obtained by subtracting the OD_492 _values of the same serum to GST alone. The results were expressed as Index of Reactivity (IR = OD_492 _values of test sample divided by the value of the cutoff). Cut-off points were set at three standard deviations above the mean OD_492 _of sera from 15 individuals, unexposed to malaria, from the city of São Paulo. Values of IR ≥ 1.0 were considered as positive.

#### Determination of antigen-specific antibodies of distinct IgG and IgM subclasses by ELISA

ELISA was performed using subclass-specific mouse anti-human IgG or anti-human IgM as the second-step reagent. These mAbs recognize human IgG1, IgG2, IgG3 or IgG4 or IgM (Sigma, Saint Louis, USA) and were diluted 1:3,000 (anti-IgG1), 1:1,000 (anti-IgG2 and anti-IgG3), 1:500 (anti-IgG4) or 1:10,000 (anti-IgM) in PBS-5% non-fat milk. After 1 h incubation at room temperature, plates were washed and peroxidase labeled anti-mouse heavy and light chain IgG (Sigma, Saint Louis, USA) was added to a final concentration of 1:5,000. Only serum samples that had IgG specific for the recombinant protein VIR were tested for IgG subclass. Each serum was tested in duplicate and the OD_492 _values were averaged. Cut-off points were calculated as described above.

### Peripheral blood mononuclear cells (PBMC) proliferation assay

The proliferation assays were performed as described elsewhere [21]. Blood samples were collected in heparinized tubes and transported to the laboratory within 20 h. After diluted with the same volume of PBS, PBMC were isolated on Ficoll-Paque (Amersham Biosciences, Uppsala, Sweden) by centrifugation. The PBMC at the interface were collected, washed three times in PBS, and ressuspended in complete media. Viable PBMC counts were made under a phase-contrast microscope by the trypan blue dye exclusion test. The complete media was RPMI 1640 medium (Invitrogen, Auckland, New Zealand) supplemented with 10% normal human serum, 2 mM L-glutamine, 10 mM Hepes, 0.2% sodium bicarbonate, 5 × 10^-5 ^M 2-mercaptoethanol, 1 mM sodium pyruvate, 1 mM non-essential amino acid solution, 100 U/ml penicilin and streptomycin. A total of 2.0 × 10^5 ^cells in 200 μl of culture media were added to each well of a flat bottom 96-well plate (Corning Co., NY). Each recombinant protein VIR, MSP1_19 _or GST was added in 20 μl. The final concentration of recombinant proteins or GST was 2 and 10 μg/ml. Because of the antigen limitation, only one recombinant protein representing each subfamily VIR was used (A4, B10, C1, and E5). The assay was performed in triplicate cultures and one of these triplicates contained only culture medium. Concanavalin A (Sigma, Saint Louis, USA) was used as positive control in all the experiments. Cultures were incubated in a humid environment, at 37°C, in a 5% CO_2 _atmosphere for 5 days. Subsequently, 25 μl of complete medium containing 1 μCi of [^3^H]-thymidine (Amersham Bioscences, Buckinghamshire, England) was added to the cultures. After 20–24h, cultures were harvested with a semi-automatic cell harvester, and radioactivity was determined in a liquid scintillation counter. The arithmetic mean cpm for each set of triplicate wells was calculated, and the stimulation indices (SI) were determined as the arithmetic mean cpm of recombinant protein-stimulated culture divided by the arithmetic mean cpm of the GST-stimulated culture to VIR proteins or medium alone to recombinant protein MSP1_19_. The background counts for GST varied from 264 to 2,126 cpm.

### Statistical analysis

Differences between proportions of responders were analysed using the Chi-square test. Mann-Whitney test was used to compare the median of the Index of Reactivity (IR) among groups. The significance level was set at *P *< 0.05.

## Results

### Purification of recombinant VIR proteins

**Figure 1:**
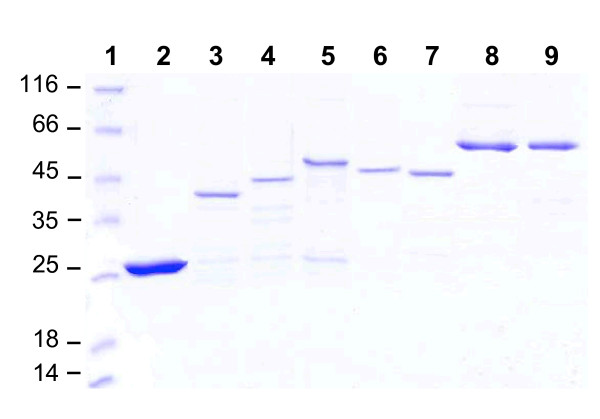
**SDS-PAGE analysis of the purified recombinant proteins corresponding to the different VIR subfamilies**. Bacterial recombinant VIR proteins were expressed and purified as described in the Methods section. Lanes, 1: Molecular mass marker; 2: GST; 3: VIR-A4; 4: VIR-B10; 5: VIR-C1; 6: VIR-C2; 7: VIR-C16; 8: VIR-E5; 9: VIR-E8.

Previous studies by us failed to solubilized GST-VIR tags [4]. In the present study, it was established that low expression temperature (18°C) and high concentrations of IPTG cooperatively improved the solubility of GST-VIR recombinant proteins. Using this strategy, soluble antigens were purified by GST affinity chromatography. Figure 1 shows the SDS-PAGE analysis of the purified recombinant proteins performed under denaturing conditions in the presence of the reducing agent 2-ME. Each protein migrated with apparent molecular weights of 42 kDa (VIR-A4), 47 kDa (VIR-B10), 52 kDa (VIR-C1 and VIR-C2), 54 kDa (VIR-C16), 59 kDa (VIR-E5 and VIR-E8).

### Recognition of recombinant VIR proteins and two merozoite surface antigens of *P. vivax *(AMA-1 and MSP1_19_) by antibodies of individuals with patent vivax malaria

Serum antibodies (IgM, IgG and IgG subclasses) from individuals living in different endemic areas for malaria in the north of Brazil were tested for recognition of VIR proteins. Initially, 200 serum samples collected from individuals with patent infection were tested by ELISA, using as antigens seven GST-fusion proteins representing four VIR subfamilies (A, B, C, and E). The results show that a total of 49.0% of subjects presented antibodies (IgM plus IgG) to at least one of the recombinant proteins representing VIR antigens indicating that these proteins are immunogenic during natural human infections. The frequency of individuals that presented IgG antibodies to at least one of the recombinant proteins VIR was 26.0%. Table 1 shows that the frequency of responders against each recombinant protein VIR ranged from 2.0% (protein VIR-C16) to 17.5% (protein VIR-C2). None of the seven VIR proteins tested was immunodominant. However, when the prevalence of individuals that presented IgG antibodies to each VIR subfamily was analysed, the prevalence of antibodies against VIR-C subfamily was significantly higher than the prevalence against other subfamilies (*P *< 0.05, Chi-Square test). Recombinant proteins AMA-1 and MSP1_19 _were also used to compare the reactivity of serum samples. The frequency of individuals with IgG antibodies to AMA-1 and MSP1_19 _was 57.0% and 90.5%, respectively (Table 1).

**Table 1:** Prevalence of the antibody response in 200 individuals with patent *Plasmodium vivax *malaria

	**Number of positive sera (%)**	
	
**Recombinant protein**	**IgG**	**IgM**
**VIR-A4**	22 (11.0)	36 (18.1)
**VIR-B10**	22 (11.0)	18 (9.0)
**VIR-C1**	23 (11.5)	12 (6.0)
**VIR-C2**	35 (17.5)	22 (11.0)
**VIR-C16**	4 (2.0)	30 (15.1)
**VIR-E5**	20 (10.0)	26 (13.1)
**VIR-E8**	5 (2.5)	30 (15.1)
**AMA-1**	114 (57.0)	ND
**MSP1**_**19**_	181 (90.5)	ND

The percentage of responders was calculated as described in the Methods section. Serum samples were tested at a dilution of 1:100. The cut-off values for each recombinant protein were: VIR-A4, 0.166; VIR-B10, 0.121; VIR-C1, 0.119; VIR-C2, 0.136; VIR-C16, 0.183; VIR-E5, 0.116; VIR-E8, 0.251; AMA-1, 0.341; MSP1_19_, 0.192 (for IgG). VIR-A4, 0.165; VIR-B10, 0.099; VIR-C1, 0.204; VIR-C2, 0.190; VIR-C16, 0.138; VIR-E5, 0.143; VIR-E8, 0.150 (for IgM)

The frequencies of individuals who responded to recombinant proteins VIR were significantly lower than the frequency of responders to AMA-1 and MSP1_19 _(*P *< 0.05, Chi-Square test). When the IR values from individual serum samples were compared, values obtained for both were higher than those observed for VIR (data not shown, *P *< 0.05, Mann-Whitney). A schematic diagram of the IgG antibody response against recombinant VIR proteins, in each individual with patent *P. vivax *malaria, is shown in Additional File 1. In addition, the IgM prevalence against each recombinant protein VIR was also evaluated. A total of 29.6% of subjects recognized at least one of the recombinant proteins VIR. As can be seen in Table 1 the frequency of responders against each recombinant protein VIR ranged from 6.0% (protein VIR-C1) to 18.1% (protein VIR-A4).

Because the subclass of IgG produced in response to a given antigen may determine the function of the antibody, the IgG subclasses were estimated by subclass-specific ELISA using as antigen recombinant proteins VIR. No statistically difference was observed in IgG subclass distribution of antibodies, except to VIR-C whose antibodies are mainly IgG1 subclass (Table 2).

**Table 2:** Frequency of malaria exposed individuals with antibodies of distinct IgG subclass specific for recombinant proteins VIR

	**% of positive sera**			
	
**IgG**	**VIR-A**	**VIR-B**	**VIR-C**	**VIR-E**
**Subclass**				
**IgG1**	9.5	4.8	38.6	15.0
**IgG2**	14.3	0	13.6	20.0
**IgG3**	14.3	4.8	15.9	25.0
**IgG4**	14.3	0	11.4	0

The percentage of responders was calculated as described in the Methods section. The ELISA was performed using subclass-specific, anti-human IgG1, IgG2, IgG3 or IgG4 and serum samples at a dilution of 1:25. Only serum samples that had total IgG specific for proteins VIR were tested in this assay. The cut-off values were defined as the mean plus 3 standard deviation values obtained from 20 uninfected control sera.

**Figure 2:**
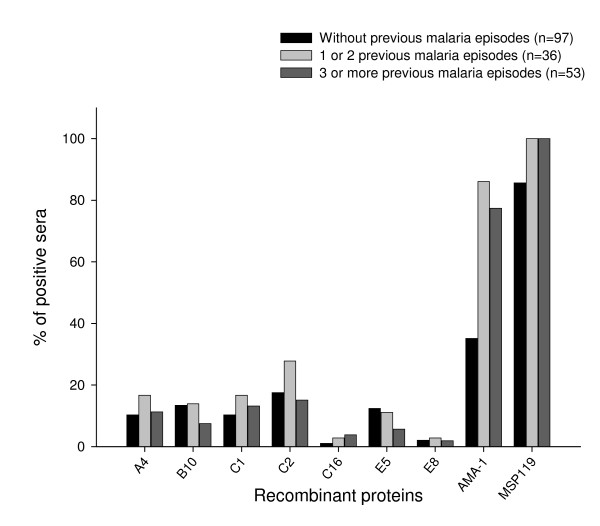
**Association between the percentage of responders that recognize each recombinant protein and the number of previous episodes malaria**. A total of 186 individuals were grouped according to the number of times they had episodes of *P. vivax *malaria. The cut-off values were the same as used for Table 1. There was a significant increase in the percentage of individuals containing IgG anti-AMA-1 in the groups with previous malaria episodes when compared with individuals without previous episodes of the disease (*P *< 0.05, Chi-Square test). No statistically significant difference was observed among the frequencies of sera from individuals containing IgG antibodies anti-VIR and anti-MSP1_19 _in the different groups of individuals (*P *> 0.05, Chi-Square test).

In order to determine whether there was a correlation between the frequency of IgG antibodies and episodes of *P. vivax *infection, the sera of the 186 individuals were separated according to the previous number of malaria episodes. Individuals were divided in three groups: i) primary-infected, individuals with no previous malaria episodes; ii) individuals with 1 or 2 previous malaria episodes; and iii) individuals with ≥ 3 previous malaria episodes. The frequency of responders to recombinant proteins VIR did not increase according to the number of previous *P. vivax *malaria episodes (Figure 2, *P *> 0.05, Chi-Square test) which confirms recently obtained data [4]. In contrast, the frequency of serum samples containing IgG anti-AMA-1 was significantly higher in groups ii and iii, that had previous malaria episodes when compared with the group without previous malaria episodes (*P *< 0.05, Chi-Square test). This result suggests that the infection provides a boost to the production of antibodies specific for the AMA-1 that is not observed in the case of the VIR proteins. The frequency of individuals containing anti-MSP1_19 _IgG was high (85.6%) in the group without previous malaria episodes, confirming previous data showing that antibody response against MSP1_19 _was established after a single exposure to malaria [18,19].

### PBMC proliferative response after in vitro culture with different recombinant proteins VIR and MSP1_19_

PBMC were collected from 27 individuals recently treated for malaria infection. These cells were tested for proliferation upon culture in the presence of one representing of each VIR subfamily (A4, B10, C1, and E5), GST or MSP1_19_. Recombinant proteins or GST were tested, in triplicates, at the two distinct final concentrations of 2 and 10 μg/ml. Individuals who showed SI > 2.5 for at least one of the two concentrations of recombinant protein, when compared to PBMC cultures containing the GST alone (VIR) or medium alone (MSP1_19_), were considered as positive responders. The frequency of individuals who responded to each recombinant protein was estimated. As control, it was estimated the frequency of responders to each recombinant protein among 14 healthy subjects resident in the city of São Paulo, State of São Paulo, in the southeast of Brazil. These individuals had never had malaria or traveled to malaria-endemic areas.

**Figure 3:**
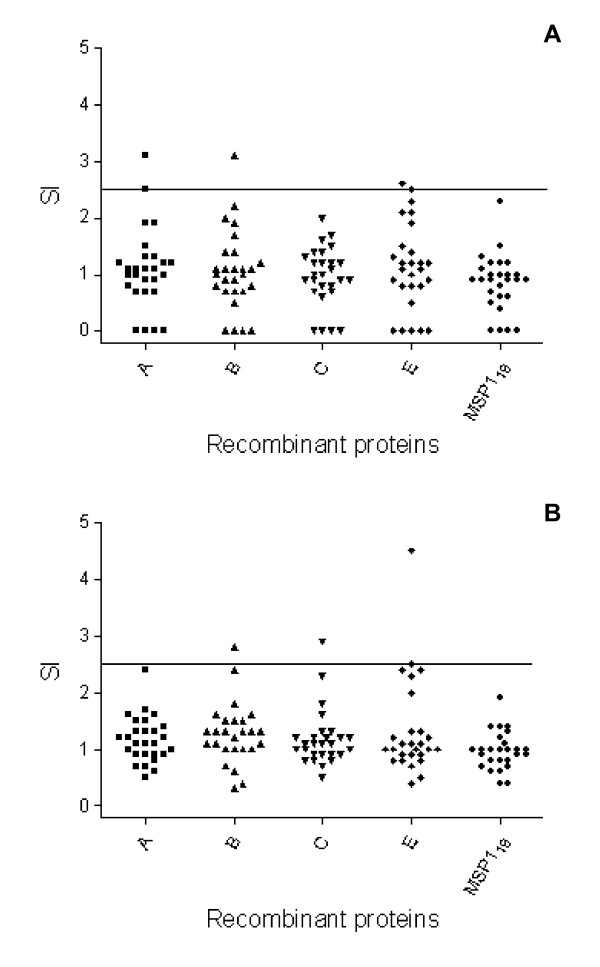
**PBMC proliferative response to recombinant proteins VIR and MSP1_19 _from individuals recently exposed to *P. vivax *malaria**. PBMC proliferation assay was performed as described in the Methods section. Each recombinant protein VIR, MSP1_19 _or GST was tested in final concentration of 2 and 10 μg/ml. Individuals were considered as positive responders when they showed SI > 2.5 for at least one of the two concentrations of recombinant protein when compared to PBMC cultures containing the GST protein alone (VIR proteins) or medium alone (MSP1_19_). Symbols represent the SI obtained after stimulation with each antigen at the final concentration of 2 μg/ml (A) or 10 μg/ml (B). The horizontal lines represent the SI = 2.5. Individuals never exposed to malaria did not respond to antigen stimulation (data not shown). PBMC were also stimulated with Con A as positive controls. The SI obtained after stimulation with this mitogen varied from 15 to 178.2 (data not shown).

The frequency of responders among individuals exposed to malaria was low and ranged from 3.7% (VIR-A4 and VIR-C1) to 7.4% (VIR-B10 and VIR-E5) (data not shown). PBMC obtained from 85.2% of the individuals treated for malaria failed to proliferate in response to any recombinant protein tested. The lack of response to recombinant proteins VIR and MSP1_19 _in a large number of individuals from endemic areas was not due to loss of viability in culture, because cells from all individuals responded vigorously to Con A (data not shown). None of the individuals who had never been exposed to malaria group was a responder. The proportions of responders between the two groups of individuals were compared statistically. The frequency of responders to the all recombinant proteins was not statistically different between the groups of malaria exposed and the unexposed individuals. The SI obtained after stimulation with each one of the recombinant proteins VIR and MSP1_19 _among individuals exposed to malaria is presented in Fig. 3. As one can see, the positive responders to VIR proteins had SIs between 2.6 and 4.5. No individual of the exposed group was responder to MSP1_19_.

## Discussion

In the present study, the serum antibody and PBMC reactivity of individuals exposed to *P. vivax *malaria with recombinant proteins representing *P. vivax *variant antigens (VIR) have been characterized and compared. This effort represents the first description of the antigenic properties of the VIR antigens expressed and purified as soluble proteins from *E. coli*.

Initially, it was determined the frequency of individual serum samples that contained IgM or IgG antibodies to each of the seven recombinant proteins representing four VIR subfamilies. The results show that a considerable proportion of individuals recognized at least one of these recombinant proteins (29.6% and 26.0% for IgM and IgG, respectively), although the antibody levels (estimated by the OD) were generally low. Considering the high gene copy number per haploid genome [3] and high polymorphism of the VIR proteins in natural parasite populations [4,5], these results suggest that this protein can be highly immunogenic during natural human infections.

Among the different VIR subfamilies, the VIR-C was most frequently recognized by human IgG antibodies. The fact that three recombinant proteins representing the subfamily C (VIR-C1, VIR-C2, and VIR-C16) were used, may explain the higher frequency of responders to this subfamily. Moreover, the subfamily C is one of the most polymorphic in natural parasite isolates [4].

Although it is described that VIR proteins are immunogenic during natural infections, 51% of the individuals did not present IgM or IgG antibodies to any VIR protein. On the other hand, 70.5% of them displayed IgG antibodies to recombinant proteins representing two other *P. vivax *blood stage antigens (AMA-1 and MSP1_19_), which are extremely immunogenic during natural malaria infections and conserved in *P. vivax *Brazilian isolates [19,22]. The frequencies of responders found in this study is not very different from the frequency found in an early study performed in the endemic area of the State of Rondônia, Brazil, using 22 different recombinant proteins, representing the various VIR subfamilies (A-E) from three patients [4]. However, different from the present study, the recognition of their recombinant proteins was analysed by immunoblotting after protein purification by electroelution and the VIR-A was most frequently recognized by human IgG antibodies.

Several studies with *P. falciparum *have demonstrated that agglutinating antibodies from individuals living in malaria endemic areas are known to react with the surface of the trophozoite-infected erythrocyte [9,10,23-28]. To date, it is believed that these agglutinating antibodies are directed against PfEMP-1 [10,29]. It has been shown that immunity against PfEMP-1 variants is acquired in an age-dependent manner [10,23,24,26,27] and is associated with protection from clinical disease [9,10]. Initial studies suggested that immune adults might have cross-reactive antibodies that agglutinate diverse *P. falciparum *isolates [23]. However, later studies suggested that both variant-specific and cross-reactive antibodies against PfEMP-1 might be elicited during natural infection with *P. falciparum *[30]. Very little information is available regarding the prevalence of antibodies in individuals from endemic areas against recombinant proteins representing specific domains of the PfEMP-1 [31].

A second antigen belonging to the largest known family of variable infected erythrocyte surface-expressed proteins, the rifin proteins, is also immunogenic in natural infections. High levels of specific antibodies against recombinant rifin proteins were detected in the majority of the adults living in an area of intense malaria transmission [32,33]. Despite the high degree of diversity between *rif *sequences and the high gene copy number, it appears that *P. falciparum *infections can induce antibodies that cross-react with several variant rifin molecules in many parasite isolates in a given community, and the immune response is most likely stable over time in a hyperendemic area [32].

It was observed an equally distributed prevalence of IgG subclass against each of the different recombinant proteins VIR, except to VIR-C, whose antibodies are mainly IgG1 subclass. Only a few studies have addressed the IgG subclass distribution of VSA-specific antibody responses in general. These studies suggested that IgG1 is the dominant subclass involved in the VSA-specific IgG response in adults [34,35]. However, two other studies indicated that IgG3 is the dominant subclass in children [36] or that IgG3 and IgG2 are the dominant subclasses in adults, while in children IgG3 and IgG4 are the dominant subclasses [37].

Because the localities included in this study are considered as hypoendemic areas, it is not possible to establish any correlation among antibody responses against malaria proteins and age. To overcome this problem, an attempt was made to determine the correlation between antibody IgG responses to each recombinant protein VIR and number of previous malaria episodes. In contrast to the observations made to PfEMP-1 and rifin, this analysis, using a significant number of sera from primary-infected and multiple-infected individuals, showed no association between presence of anti-VIR IgG antibodies and malaria exposure. This data extended recent results obtained with a smaller number of sera from individuals from Rondônia State [4]. These results may be explained by the fact that *vir *genes representing at least two different subfamilies are transcribed concomitantly during mature stages by individual parasites and the large repertoire of VIR proteins found in natural parasite populations.

Also relevant is the observation that cross-reactive epitopes between the antigens encoded by the different *vir *subfamilies are undetectable after repeated infections. In agreement with this observation, recent attempts to demonstrate the cross-recognition within a subfamily and across subfamilies by inhibition ELISA assay failed (data not shown). It is possible that cross-reactive antibodies that are not generated by natural infections can be elicited by immunization with recombinant proteins. In fact, in ongoing studies, mice immunized with different recombinant proteins generated these cross-reactive antibodies to distinct VIR subfamilies (Oliveira and Soares, unpublished).

In the second part of the study, it was examined whether VIR antigens elicited an in vitro proliferative response of PBMC obtained from human subjects recently treated for *P. vivax *infection and normal healthy individuals. PBMC of only 14.8% of the individuals were able to proliferate to recombinant proteins VIR and when there was a T cell response, it was generally low in magnitude. Although the results suggests that the PBMC response to these recombinant proteins is negligible in *P. vivax *infections, it is impossible to rule out that they may reflect the fact that PBMC response is more specific than antibody response or that a larger number of individual proteins should be surveyed.

There are no studies of T cell responses to VIR antigens to be compared to these data. T cell responses in the field have been described elsewhere for PfEMP-1. CD4 T cell response to conserved regions of PfEMP-1 was significantly greater in malaria-exposed individuals than in unexposed Europeans, which suggests that these regions contain peptides recognized by T cells [38,39].

## Conclusion

Soluble recombinant proteins representing distinct VIR antigens subfamilies were generated and they may be useful tools to perform a number of immuno-epidemiological studies in malaria-endemic areas. Also, this study provides evidence that there is a low frequency of individuals responding to each VIR antigen in endemic areas of Brazil, a fact that may explain the host susceptibility to new episodes of infection.

## Authors' contributions

TRO and CFB generated the recombinant plasmids containing the *vir *genes. TRO purified the recombinant VIR proteins and carried out all immunological studies. MCSJ purified the recombinant protein His_6_-MSP1_19_. HADP participated in the design of the study and drafted the manuscript. ISS conceived the study, and participated in all aspects of its design, execution, coordination and manuscript preparation. All authors read and approved the final manuscript.

## Supplementary Material

Additional File 1**Schematic diagram of the IgG antibody response against recombinant VIR proteins in each individual with patent *P. vivax *malaria**. The antibody level was expressed as Index of Reactivity calculated as described in the Methods section. Values of IR ≥ 1.0 were considered as positive. Individuals primary-infected are shown in bold and underlined. Individual responses were categorized as follows: negative individuals (), individuals with IR between 1 to < 2.5 (), individuals with IR between 2.5 to < 5.0 () and individuals with IR ≥ 5.0 ().Click here for file

## References

[B1] Mendis K, Sina BJ, Marchesini P, Carter R (2001). The neglected burden of *Plasmodium vivax *malaria. Am J Trop Med Hyg.

[B2] Secretaria de Vigilância em Saúde-SVS, Ministério da Saúde. http://www.saude.gov.br.

[B3] Del Portillo HA, Fernandez-Becerra C, Bowman S, Oliver K, Preuss M, Sanchez CP, Schneider NK, Villalobos JM, Rajandream MA, Harris D, Pereira da Silva LH, Barrel B, Lanzer M (2001). A superfamily of variant genes encoded in the subtelomeric region of *Plasmodium vivax*. Nature.

[B4] Fernandez-Becerra C, Pein O, Oliveira TR, Yamamoto MM, Cassola AC, Rocha C, Soares IS, de Bragança-Pereira CA, Del Portillo HA (2005). Variant proteins of *Plasmodium vivax *are not clonally expressed in natural infections. Mol Microbiol.

[B5] Merino EF, Fernandez-Becerra C, Durham AM, Ferreira JE, Tumilasci VF, d'Arc-Neves J, da Silva-Nunes M, Ferreira MU, Wickramarachchi T, Udagama-Randeniya P, Handunnetti SM, del Portillo HA (2006). Multi-character population study of the *vir *subtelomeric multigene superfamily of *Plasmodium vivax*, a major human malaria parasite. Mol Biochem Parasitol.

[B6] Janssen CS, Phillips RS, Turner CM, Barrett MP (2004). *Plasmodium *interspersed repeats: the major multigene superfamily of malaria parasites. Nucleic Acids Res.

[B7] Kaviratne M, Fernandez V, Jarra W, Cunningham D, Galinski MR, Wahlgren M, Preiser PR, Craig A, Scherf A (2003). Antigenic Variation in *Plasmodium falciparum *and other *Plasmodium *species. Antigenic Variation.

[B8] Del Portillo HA, Lanzer M, Rodríguez-Malaga S, Zavala F, Fernandez-Becerra C (2004). Variant genes and the spleen in *Plasmodium vivax *malaria. Int J Parasitol.

[B9] Marsh K, Otoo L, Hayes RJ, Carson DC, Greenwood BM (1989). Antibodies to blood stage antigens of *Plasmodium falciparum *in rural Gambians and their relation to protection against infection. Trans R Soc Trop Med Hyg.

[B10] Bull PC, Lowe BS, Kortok M, Molyneux CS, Newbold CI, Marsh K (1998). Parasite antigens on the infected red cell surface are targets for naturally acquired immunity to malaria. Nat Med.

[B11] Dodoo D, Staalsoe T, Giha H, Kurtzhals JA, Akanmori BD, Koram K, Dunyo S, Nkrumah FK, Hyiid L, Theander TG (2001). Antibodies to variant antigens on the surfaces of infected erythrocytes are associated with protection from malaria in Ghanaian children. Infect Immun.

[B12] Tebo AE, Kremsner PG, Piper KP, Luty AJ (2002). Low antibody responses to variant surface antigens of *Plasmodium falciparum *are associated with severe malaria and increased susceptibility to malaria attacks in Gabonese children. Am J Trop Med Hyg.

[B13] Abdel-Latif MS, Dietz K, Issifou S, Kremsner PG, Klinkert MQ (2003). Antibodies to *Plasmodium falciparum *Rifin proteins are associated with rapid parasite clearance and asymptomatic infections. Infect Immun.

[B14] Smith JD, Chitnis CE, Craig AG, Roberts DJ, Hudson-Taylor DE, Peterson DS, Pinches R, Newbold CI, Miller LH (1995). Switches in expression of *Plasmodium falciparum var *genes correlate with changes in antigenic and cytoadherent phenotypes of infected erythrocytes. Cell.

[B15] Fernandez V, Hommel M, Chen Q, Hagblom P, Wahlgren M (1999). Small, clonally variant antigens expressed on the surface of the *Plasmodium falciparum *-infected erythrocyte are encoded by the *rif *gene family and are the target of human immune responses. J Exp Med.

[B16] Kyes SA, Rowe JA, Kriek N, Newbold CI (1999). Rifins: A second family of clonally variant proteins expressed on the surface of red cells infected with *Plasmodium falciparum*. Proc Natl Acad Sci USA.

[B17] Soares IS, Cunha MG, Silva MN, Souza JM, Del Portillo HA, Rodrigues MM (1999). Longevity of naturally acquired antibody responses to the N and C-terminal regions of *Plasmodium vivax *merozoite surface protein 1. Am J Trop Med Hyg.

[B18] Rodrigues MHC, Cunha MG, Machado RLD, Ferreira-Jr OC, Rodrigues MM, Soares IS (2003). Serological detection of *Plasmodium vivax *malaria using recombinant proteins corresponding to the 19-kDa C-terminal region of the merozoite surface protein-1. Malar J.

[B19] Rodrigues MHC, Rodrigues KM, Oliveira TR, Comodo AN, Rodrigues MM, Kocken CH, Thomas AW, Soares IS (2005). Antibody response of naturally infected individuals to recombinant *Plasmodium vivax *apical membrane antigen-1. Int J Parasitol.

[B20] Cunha MG, Rodrigues MM, Soares IS (2001). Comparison of the immunogenic properties of recombinant proteins representing the *Plasmodium vivax *vaccine candidate MSP1_19 _expressed in distinct bacterial vectors. Vaccine.

[B21] Soares IS, Levitus G, Souza JM, Del Portillo HA, Rodrigues MM (1997). Acquired immune responses to the N- and C-terminal regions of *Plasmodium vivax *merozoite surface protein 1 in individuals exposed to malaria. Infect Immun.

[B22] Soares IS, Barnwell JW, Ferreira MU, Cunha MG, Laurino JP, Castilho BA, Rodrigues MM (1999). A *Plasmodium vivax *vaccine candidate displays limited allele polymorphism, which does not restrict recognition by antibodies. Mol Med.

[B23] Marsh K, Howard RJ (1986). Antigens induced on erythrocytes by *P. falciparum *: expression of diverse and conserved determinants. Science.

[B24] Forsyth KP, Philip G, Smith T, Kum E, Southwell B, Brown GV (1989). Diversity of antigens expressed on the surface of erythrocytes infected with mature *Plasmodium falciparum *parasites in Papua New Guinea. Am J Trop Med Hyg.

[B25] Newbold CI, Pinches R, Roberts DJ, Marsh K (1992). *Plasmodium falciparum *: the human agglutinating antibody response to the infected red cell surface is predominantly variant specific. Exp Parasitol.

[B26] Iqbal J, Perlmann P, Berzins K (1993). Serological diversity of antigens expressed on the surface of erythrocytes infected with *Plasmodium falciparum*. Trans R Soc Trop Med Hyg.

[B27] Bull PC, Lowe BS, Kortok M, Marsh K (1999). Antibody recognition of *Plasmodium falciparum *erythrocyte surface antigens in Kenya: evidence for rare and prevalent variants. Infect Immun.

[B28] Giha HA, Staalsoe T, Dodoo D, Elhassan IM, Roper C, Satti GMH, Arnot DE, Theander TG, Hviid L (1999). Nine-Year longitudional study of antibodies to variant antigens on the surface of *Plasmodium falciparum *-infected erythrocytes. Infect Immun.

[B29] van Schravendijk MR, Rock EP, Marsh K, Ito Y, Aikawa M, Neequaye J, Ofori Adjei D, Rodriguez R, Patarroyo ME, Howard RJ (1991). Characterization and localization of *Plasmodium falciparum *surface antigens on infected erythrocytes from west African patients. Blood.

[B30] Chattopadhyay R, Sharma A, Srivastava VK, Pati SS, Sharma SK, Das BS, Chitnis CE (2003). *Plasmodium falciparum *infection elicits both variant-specific and cross-reactive antibodies against variant surface antigens. Infect Immun.

[B31] Oguariri RM, Borrmann S, Klinkert MQ, Kremsner PG, Kun JF (2001). High prevalence of human antibodies to recombinant Duffy binding-like alpha domains of the *Plasmodium falciparum *-infected erythrocyte membrane protein 1 in semi-immune adults compared to that in nonimmune children. Infect Immun.

[B32] Abdel-Latif MS, Khattab A, Lindenthal C, Kremsner PG, Klinkert MQ (2002). Recognition of variant Rifin antigens by human antibodies induced during natural *Plasmodium falciparum *infections. Infect Immun.

[B33] Abdel-Latif MS, Cabrera G, Kohler C, Kremsner PG, Luty AJ, 1-95/C. Study Team (2004). Antibodies to rifin: a component of naturally acquired responses to *Plasmodium falciparum *variant surface antigens on infected erythrocytes. Am J Trop Med Hyg.

[B34] Piper KP, Roberts DJ, Day KP (1999). *Plasmodium falciparum *: analysis of the antibody specificity to the surface of the trophozoite-infected erythrocyte. Exp Parasitol.

[B35] Megnekou R, Staalsoe T, Taylor DW, Leke R, Hviid L (2005). Effects of pregnancy and intensity of *Plasmodium falciparum *transmission on immunoglobulin G subclass responses to variant surface antigens. Infect Immun.

[B36] Kinyanjui SM, Bull P, Newbold CI, Marsh K (2003). Kinetics of antibody responses to *Plasmodium falciparum *-infected erythrocyte variant surface antigens. J Infect Dis.

[B37] Cabrera G, Yone C, Tebo AE, Van Aaken J, Lell B, Kremsner PG, Luty AJ (2004). Immunoglobulin G isotype responses to variant surface antigens of *Plasmodium falciparum *in healthy Gabonese adults and children during and after successive malaria attacks. Infect Immun.

[B38] Allsopp CE, Sanni LA, Reubsaet L, Ndungu F, Newbold C, Mwangi T, Marsh K, Langhorne J (2002). CD4 T cell responses to a variant antigen of the malaria parasite *Plasmodium falciparum*, erythrocyte membrane protein-1, in individuals living in malaria-endemic areas. J Infect Dis.

[B39] Sanni LA, Allsopp CE, Reubsaet L, Sanni A, Newbold C, Chauhan VS, Langhorne J (2002). Cellular responses to *Plasmodium falciparum *erythrocyte membrane protein-1: use of relatively conserved synthetic peptide pools to determine CD4 T cell responses in malaria-exposed individuals in Benin, West Africa. Malar J.

