# Supplementary material for: Evaluation of the acquired immune responses to Plasmodium vivax VIR variant antigens in individuals living in malaria-endemic areas of Brazil
Source: Malar J. 2006 Oct 6;5:83. doi: 10.1186/1475-2875-5-83 (PMC1626480; doi:10.1186/1475-2875-5-83)
Supplement: Additional File 1 — Schematic diagram of the IgG antibody response against recombinant VIR proteins in each individual with patent P. vivax malaria. The antibody level was expressed as Index of Reactivity calculated as described in the Methods section. Values of IR ≥ 1.0 were considered as positive. Individuals primary-infected are shown in bold and underlined. Individual responses were categorized as follows: negative individuals (), individuals with IR between 1 to < 2.5 (), individuals with IR between 2.5 to < 5.0 () and individuals with IR ≥ 5.0 (). [file 1475-2875-5-83-S1.rtf]

		Recombinant VIR proteins	
		A4	B10	C1	C2	C16	E5	E8		A4	B10	C1	C2	C16	E5	E8		A4	B10	C1	C2	C16	E5	E8	
	1								68								135								
Subject number 	2								69								136								
	3								70								137								
	4								71								138								
	5								72								139								
	6								73								140								
	7								74								141								
	8								75								142								
	9								76								143								
	10								77								144								
	11								78								145								
	12								79								146								
	13								80								147								
	14								81								148								
	15								82								149								
	16								83								150								
	17								84								151								
	18								85								152								
	19								86								153								
	20								87								154								
	21								88								155								
	22								89								156								
	23								90								157								
	24								91								158								
	25								92								159								
	26								93								160								
	27								94								161								
	28								95								162								
	29								96								163								
	30								97								164								
	31								98								165								
	32								99								166								
	33								100								167								
	34								101								168								
	35								102								169								
	36								103								170								
	37								104								171								
	38								105								172								
	39								106								173								
	40								107								174								
	41								108								175								
	42								109								176								
	43								110								177								
	44								111								178								
	45								112								179								
	46								113								180								
	47								114								181								
	48								115								182								
	49								116								183								
	50								117								184								
	51								118								185								
	52								119								186								
	53								120								187								
	54								121								188								
	55								122								189								
	56								123								190								
	57								124								191								
	58								125								192								
	59								126								193								
	60								127								194								
	61								128								195								
	62								129								196								
	63								130								197								
	64								131								198								
	65								132								199								
	66								133								200								
	67								134																
